# LIVING WELL? A MATCHED ANALYSIS OF MINORITY ETHNIC AND WHITE BRITISH PEOPLE WITH DEMENTIA AND THEIR CARERS

**DOI:** 10.1093/geroni/igad104.1432

**Published:** 2023-12-21

**Authors:** Christina Victor, Catherine Quinn, Fiona Matthews, Claire Pentecost, Laura Gamble

**Affiliations:** Brunel University-London, Uxbridge, England, United Kingdom; Bradford University, Bradford, England, United Kingdom; Newcastle University, Newcastle upon Tyne, England, United Kingdom; University of Exeter, Exeter, England, United Kingdom; Newcastle University, Newcastle upon Tyne, England, United Kingdom

## Abstract

The increasing heterogeneity of the older population is reflected in the UK in an increasing number of those with dementia and carers drawn from minority ethnic groups. Data from the IDEAL dementia study is used to compare ‘living well’ among people with dementia and carers from ethnic minority groups with matched white British peers. We used a cross-sectional case-control design. Outcomes for both groups were quality of life, life satisfaction, wellbeing, loneliness and social isolation and, for carers, measures of stress, relationship quality, role captivity and caring competence. Our sample consisted of 20 people with dementia and 15 carers who self-identified as being of Indian, Pakistan, Bangladeshi, Black African, Black Caribbean or mixed ethnicity who were matched with 60 white British participants with dementia and 45 white British carers. People with dementia from minority ethnic groups had poorer quality of life (-4.74, 95% CI: -7.98 to -1.50) and higher loneliness (1.72, 95% CI: 0.78 to 2.66) scores whilst minority ethnic carers had higher stress (8.17, 95% CI: 1.72 to 14.63) and role captivity (2.00, 95% CI: 0.43 to 3.57) and lower relationship quality (-9.86, 95% CI: -14.24 to -5.48) than their white British peers. People with dementia from minority ethnic groups experience lower quality of life and caregivers higher stress and role captivity and lower relationship quality. Confirmatory research with larger samples is required to facilitate analysis of the experiences of specific minority ethnic groups and examine the factors contributing to these disadvantages.

